# Patient experiences of waiting times in standardised cancer patient pathways in Norway – a qualitative interview study

**DOI:** 10.1186/s12913-021-06679-8

**Published:** 2021-07-05

**Authors:** Marit Solbjør, Kari Sand, Bente Ervik, Line Melby

**Affiliations:** 1 Department of Public Health and Nursing, Faculty of Medicine and Health Sciences, Norwegian University of Science and Technology, 7491 Trondheim, Norway; 2 SINTEF, Department of Health Research, P.O.Box 4760 Torgården, 7465 Trondheim, Norway; 3 Department of Oncology, University Hospital of North Norway, Tromsø, Norway

## Abstract

**Objective:**

Standardised cancer patient pathways (CPP) are implemented within cancer care with an aim to ensure standardised waiting times for diagnosis and treatment. This article investigates how patients in Norway experience waiting times within a CPP.

**Methods:**

Qualitative semi-structured interviews with 19 patients who had been through CPP for breast cancer, prostate cancer or malignant melanoma in Norway.

**Results:**

Few patients knew about the term CPP but trusted that waiting times were standardised to decrease mortality. Their experiences of waiting depended on their expectations as much as the period they waited. Patients generally felt safe about the timing of treatment, but not all expectations of a rapid response from health services were met. Short waiting times were interpreted as a sign of urgency, and a change of pace between urgent action and prolonged periods of waiting were disturbing.

**Conclusions:**

Patients are comforted by knowing they are within a structured CPP that ensures rapid diagnosis and start of treatment. CPPs still need to be improved to avoid delays, allow for adaptions to patient needs, and include more information to avoid stress.

## Highlights – 3–5 bulletpoints

- CPPs provide safety and trust in timely diagnosis and treatment for patients.
- CPPs are not a guarantee for patient satisfaction with longevity of waiting.
- Changing pace between shorter and longer waiting times could cause distress when rapid action is interpreted by patients as signalling urgency.
- Experiences of waiting times within a CPP relies on patient expectations to CPP time frames.

## Introduction

Reducing the waiting time between symptom presentation and a cancer diagnosis may reduce mortality, although the association between time and survival varies between different forms of cancer [1–3]. Longer diagnostic intervals are associated with increasing 5-year mortality for colorectal, lung, melanoma, breast and prostate cancers [4]. Nevertheless, unwanted variance in waiting times between regions and hospitals have been identified in several countries (e.g., Denmark and Sweden) [4, 5]. To decrease differences in health services and reduce waiting times for cancer patients, Scandinavian countries have implemented standardised cancer patient pathways (CPP). CPPs are time-bound, standardised patient pathways describing the organisation of diagnosis and treatment, as well as communication with the patient. The Scandinavian CPPs cover the period from suspicion of cancer to the start of treatment and identifies time frames for waiting times in each phase of the pathway. Waiting time may occur during four phases of a CPP: between the first contact with a primary care provider and initiation of symptom investigation, between the initiation of symptom investigation and subsequent referral, between the referral to the specialist and first visit to the specialist; and between the referral for treatment and the commencement of treatment [5].

For some patients, waiting for diagnosis or treatment is the worst part of their cancer trajectory [1]. Becoming a cancer patient might change perceptions of time, thus, while health professionals experience time as linear, patients’ experiences might be different and concern other aspects than clock time, for instance, experiencing time as existential [6]. Thus, the waiting times for cancer patients could be as much about how the patient experiences waiting as the objective period of time [7]. In an Australian survey-based study of 146 cancer patients, almost half of the patients reported concerns about waiting times during some stage of the diagnostic and treatment pathway [8]. A Canadian study found that prostate cancer patients perceived delay as any interval longer than what the patient anticipated or found reasonable, even when those who perceived a delay did not have longer a diagnostic or treatment interval compared to those who did not [9]. A survey of breast cancer patients (*n* = 4626) found that satisfaction increased if patients experienced good communication with health personnel [10]. After the implementation of CPPs in Denmark, patients became less dissatisfied with waiting times for getting an appointment in the specialist health service [11]. They did, however, become less satisfied with the waiting time to get an appointment with a general practitioner (GP), even though this part of the process is outside the CPP—suggesting that increased public attention to waiting times due to CPPs could lead to increased expectations towards reduced waiting times in general [11].

CPPs imply that health services standardise how they organise patient trajectories. Standards and standardisation are an important part of modern healthcare [12] and concern design standards, terminology standards (such as the ICD), performance standards (such as time frames), and procedural standards (the steps that should be taken) [13]. Care pathways are tools for standardisation of the delivery of clinical care and often describe a protocol for diagnosis, treatment, and care, including the time frame which should be allowed to complete diagnostic procedures or treatment [14]. Standardisation through CPPs is meant to ensure predictability, accountability and objectivity for health services and patients [15]. The association of standardisation with objectivity [16] could lead patients to understand time frames within the CPPs as evidence-based. But, while standardisation of cancer treatment follows evidence-based guidelines, the standardisation of time frames is also based on political solutions to reduce unwanted variations in waiting times and treatment options across hospitals and regions [17]. Politicians might wish to decrease waiting times since long waiting times can lead to low patient satisfaction [10, 18] or decreased trust in health services. An Australian study did, however, find that patients waiting for an appointment in public hospitals accepted the waiting times without losing trust, since they knew resources were limited [19].

In Norway, 28 CPPs based on the national guidelines for cancer diagnosis and treatment [20] were implemented in 2015. The Norwegian public health services are based on universal health insurance and state ownership of hospital trusts, allowing standardisation of health services across the nation. The Norwegian CPPs, like other standardised pathways, are organised in phases that should be completed within a predefined time, based on the type of cancer. The goal is to ensure compliance with the overall target time in the pathway for 70 % of the patients, but target times are normative and not legally binding. A CPP begins when the hospital receives a referral for a CPP based on a well-founded suspicion of cancer, typically from the patient’s general practitioner (GP), and it ends when the initial treatment starts [20].

Such standardisation could potentially influence how patients experience waiting times during their cancer trajectory, but no previous studies have investigated how patients experience waiting times within CPPs in Norway. The aim of this study was to investigate how patients experienced waiting times within standardised CPPs.

## Methods

This was a retrospective qualitative interview study with 19 patients who had been through a CPP for breast cancer, prostate cancer, or malignant melanoma in Norway. We chose a qualitative method to explore a phenomenon where little was known beforehand and to allow individuals who had been through a CPP to define themselves what had been important to them during their trajectory, instead of, for instance, using a deductive survey study. A retrospective design was chosen to ensure that patients had completed the CPP (i.e., until at least beginning of treatment) before the interview.

### Recruitment and sample

Participants were recruited from three hospitals across Norway. The north hospital recruited patients with breast cancer and melanoma. The central hospital recruited patients with breast cancer, prostate cancer, and melanoma, and the south hospital recruited patients with prostate cancer. All patients who had been through one of these CPPs and had begun or completed their treatment were eligible for inclusion. A nurse at each hospital identified patients who fitted the inclusion criteria and contacted them with information about the study. If willing to participate, they could contact the researcher at a given telephone number to be included in the study. Staff at the hospital did not know which of the patients had chosen to participate. All patients with melanoma and men with prostate cancer at the south hospital were contacted through a letter with study information. All breast cancer patients and prostate cancer patients at the central hospital received information about the study from a nurse when having an appointment at the clinic. One participant was self-recruited after hearing about the study in an informal setting.

Participants were aged from 40 to 75 years, and all had received a cancer diagnosis in the timespan 6 weeks to 1 year before the interview. Five men participated in the study, of which four had prostate cancer and one had melanoma. Of the 14 women, two women had melanoma and 12 women had breast cancer. The distribution of participants across hospitals and cancer types is found in Table 1.

**Table 1:** Participants’ form of cancer and recruitment hospital

Hospital	North	Central	South	Sum
Breast cancer	5	7	--	**12**
Prostate cancer	--	3	1	**4**
Melanoma	1	2	--	**3**
Sum	**6**	**12**	**1**	**19**

### Data collection

Individual interviews were conducted by three researchers with substantial experience with qualitative interviewing, using a preset semi-structured interview guide. The interview guide included questions on a range of patient experiences with CPP, such as how they were met by the health services, information, shared decision-making, and waiting time. Ten interviews were carried out face-to-face in a meeting room at the university or hospital, one patient was interviewed at home, and one patient was interviewed in a public building. Seven interviews were done by phone due to long travel distances. Interviews lasted 20–75 min.

### Data analysis

Data were analysed through thematic analysis inspired by phenomenology [21]. First, we read all interviews to get a sense of the main themes. Since the interview guide had questions on several issues on patients’ experiences with CPP, we sorted the meaning units related to waiting times from each interview in order to investigate experiences of waiting times. For the purpose of the present article, our analysis focused solely on these data, which were subject to meaning condensation. Meaning units were coded and themes sorted by using NVIVO. After identifying the meaning content, we searched for the central themes in the meaning units and classified these together across the interviews. First, we structured our findings according to the timeline of the experiences, classifying the data in three periods of waiting (before the examination, waiting for the result, and waiting for the treatment). Through this process, all authors participated in analysis meetings where we discussed the different interpretations of data and the credibility of the central themes. The interviews showed contradictory experiences among the different participants, and we were careful to ensure that different voices were included in the final analysis. During the analytical process, we identified themes across the phases of the timeline, such as safety and worries, expectations and disappointments. Through exploring the meaning units in light of the research question, we identified the three main themes, which are presented in the Results section.

### Ethics

The study was approved by the Norwegian Centre for Research Data (NSD). All participants gave written informed consent after receiving information on how their data would be securely processed and their anonymity ensured. Participation in the study was voluntary, and patients were informed that their treatment would not be influenced by their choice of participation. Personal data was anonymised during transcriptions, and no identifiable information is provided in the presentation of the results.

## Results: experiences of waiting time in the CPP depended on patients’ expectations

The main theme of our results is “Experiences of waiting time in the CPP depended on patients’ expectations” and comprise the subthemes “Feeling safe about waiting times within the CPP”, “Not all expectations were met” and “Changing pace through the CPP caused worry”. These results present patient experiences with waiting times from three CPPs in Norway. All participants with prostate cancer and malignant melanoma, as well as most women with breast cancer, had entered the CPP through a referral from their GP. Some women with breast cancer had entered the CPP through the national breast cancer screening programme, and one woman contacted the hospital directly. Patients experienced waiting within the CPP in three phases: waiting for a diagnostic test, waiting for the result after having the diagnostic test, and waiting for treatment after having the diagnosis. Many of the women with breast cancer waited only a few days between their visit to the GP and their appointment at the hospital. For men with prostate cancer and persons with malignant melanoma, the waiting time was up to a month. After having the diagnostic test, some participants had received the diagnostic result on the day of the test, while others waited up to 3 weeks. After having the cancer diagnosis, patients experienced waiting for surgery or other treatment start-up from a couple of days up to a month. Despite understanding the timing of treatment as evidence-based (and therefore not posing a threat to their prognosis), these participants had varied experiences while waiting for cancer treatment within the CPP.

### Feeling safe about waiting times within the CPP

Those who had heard about CPP knew it implied target times for diagnosis and treatment. For these patients, CPPs meant safety and predictability, and they trusted to have a diagnosis in due time and did not worry about being left behind by the hospital due to other priorities. Two breast cancer patients had experienced being transferred to another diagnostic unit when their first assigned hospital could not guarantee meeting the target times of the CPP. Thus, in their experience, health services went to lengths to assure timely cancer diagnosis.

*It was quick. I understood that, in the CPP, you had the right to have treatment within a week. But for me, it only took half the time. (Participant 17, malignant melanoma)*

Experiencing a short waiting time between diagnosis and the beginning of treatment gave patients a feeling of being prioritised. It felt safe to be in the CPP after being diagnosed with cancer, since it gave a clear direction. Some participants interpreted the CPP as providing guidelines for treatment. For others, the CPP meant that they had rights with regards to time frames for treatment.

*It went really fast, and I felt safe from knowing that I was within a standardised pathway. In the sense that they have time frames to keep, and they have a handbook for how to do stuff. (Participant 1, breast cancer)*

Patients experienced their waiting times as quick when surpassing their expectations for timing. A quick answer on the results of diagnostic tests was welcomed by most patients, but they varied in what they saw as the cause of their waiting time expectations. Some had been told by health personnel that their test result would be ready within a certain time frame. Others could not explain where their expectations had come from. Regardless of origin, having the test result earlier than expected was experienced as positive, while experiencing a breach of expectations was disturbing.

### Not all expectations were met

Some patients were dissatisfied with the time it had taken to enter the CPP. They had expected that they would be rapidly referred to specialist health services but met obstacles to their wishes due to how the inclusion into the CPP was organised. One patient with melanoma and men who had done PSA screening reported having to push their GP into referring them to the CPP. For them, the waiting time before having diagnostic testing was perceived as too long. Patients who opted for nonstandardised solutions also reported not having their expectations met. Not wishing to follow the standard diagnostic test regime or preferring a different hospital than the one automatically scheduled by the health service system led to what patients experienced as a delayed entrance into the CPP. One woman who was reluctant towards having a specific diagnostic test had to wait several months before entering the CPP. In the end, she was referred to a private clinic that could offer her the desired examination, and subsequently, she was included in a CPP. Those who opted to choose a specific hospital were told that this could lead to a delay of the standardised time frames of the CPP. One woman with breast cancer was disappointed that the health services denied her requests, but she accepted waiting longer to have her hospital of choice.

Being in a CPP, waiting for a potential cancer diagnosis was stressful and patients experienced moments of despair and disbelief in the system’s efficiency. In retrospect, memories of these moments framed participants’ views on the importance of being well-informed about waiting times and standardised time frames.

*Time runs slow when you are waiting and also when you are waiting for the test result, of course, but it takes as long as it takes. They have to analyse the sample, and they have to do it properly and be sure [about the result]. But, then you speculate “does it really take 3 weeks to analyse that test?”. […] But they just told me that “you’ll have a new appointment, in 3 weeks’ time and then we’ll have the results.” I think that I wouldn’t be calmer if I had to wait for a phone call. So, I guess it is the smart way that they tell you “you’ll have your answer when you return, we won’t call you before then”. Because if they had, I would have checked my phone all the time, right? (Participant 4, breast cancer)*

Waiting time stress could be mitigated by having healthcare workers communicate with the patient when a delay happened. One patient with prostate cancer and one patient with breast cancer both reported the delay as acceptable when being contacted by their hospital with information of a new time frame.

*First, they told me it would be a week, but when the test results were delayed, they called me and told me that “I am sorry that we haven’t received your results. I know you are waiting for them, so I just want to inform you that it might be a few days more to wait”. This was good to know because you are on the alert. (Participant 3, breast cancer)*

Regardless of knowledge about CPPs, information from healthcare personnel about expected time frames led to waiting being tolerable.

### Changing pace through the CPP caused worry

Despite being in a CPP, not all participants had expected to have a cancer diagnosis. Some told of having few thoughts about cancer while waiting for the test result. Not foreseeing a cancer diagnosis had, according to them, freed them of cancer worries during the waiting period. But, assuming that the test would be negative (i.e., not having cancer), having the result and a hospital appointment within a short time frame was disturbing, as it gave the impression that their condition demanded quick action.

*Well, I was so sure everything was fine. But, when I came to the clinic on that Tuesday, that’s when I was told. Or, I had already understood it when I had to go to [the city hospital] to get my test results. I realised there was something wrong. (Participant 6, breast cancer)*

Some participants experienced the process as passing too quickly after having a cancer diagnosis. They felt that they needed time to be mentally prepared before having surgery or attend to practical arrangements before entering a period of sick leave. For participants who had worried about having cancer from the initiation of the CPP, a rapid test result was frightening when interpreting a short waiting time as a sign of urgency. When receiving a cancer diagnosis, these participants interpreted the actions of health services as related to their individual diagnosis and prognosis and not as a standardised response from health services that had implemented CPP.

*I received my call from the hospital already on the following Monday. It scared me because this had to be serious, since they wanted me in so soon. It was reassuring too, because then I knew what the next step would be. […] Once you say the word “cancer”, you think the worst, right? I immediately imagined cancer spreading rapidly. (Participant 4, breast cancer)*

The changing pace of health services during the CPP was disturbing for participants. A perceived long waiting period before a diagnostic test, followed by sudden urgency when the test result showed cancer, was frightening. Changing between long waiting and rapid action led to the reinterpretation of the first waiting period as too long, or as a waste of time, which could have contributed to a better prognosis. In our data, it was particularly men with prostate cancer who experienced waiting as a waste of time. They had to move between a local hospital and a regional hospital, which had their own CPP routines, despite previous examinations at the local hospital. For these men, such a transfer was perceived as a delay and unnecessary waiting time, and they blamed it on the standardisation of the CPP. In their interpretation, the organisation of the CPP added extra time before having surgery.

*I can’t see why it should take a full month to see a surgeon when I already had been to a specialist. It was nonsensical to lose that month, completely unnecessary to lose four weeks. […] They might as well have provided surgery as they were talking to me that day. (Participant 8, prostate cancer)*

Several patients also felt that the action provided towards cancer was not quick enough when they knew that cancer was present within their body. Even when assuming that the timing within the CPP was based on advice that would not put them at risk for higher mortality, the need for action led to a difficult waiting period. Thus, a change of pace during the CPP was frightening or disturbing regardless of whether it comprised a long wait for a diagnosis and then rapid action for treatment or the opposite—rapid diagnosis followed by a longer wait for the start of treatment.

## Discussion

This study has investigated how patients experience waiting times for cancer diagnosis and treatment within three CPPs. Patients in the study felt safe from being in a CPP, as they trusted that standardised waiting times were evidence-based and therefore not endangering their prognosis. Also, they thought they would not be forgotten by the hospital. The patients’ experiences of waiting times—across the phases of CPP—relied on their expectations. Not all expectations were met, and consequently, some patients had seen what they experienced as a delay. However, it was the changing pace through the CPP that caused them to worry, since rapid action at one point led them to see their diagnosis as urgency.

Patients may experience concern about waiting times at some stage of the diagnostic and treatment pathway [8]. For patients, predictability is important, since their experiences of waiting times are often based on expectations [7] and not solely on the length of diagnostic or treatment intervals [9]. CPP standardisation is meant to ensure predictability, accountability and objectivity for health services and patients [15]. In the present study, participants felt safe about waiting times when they knew them to be standardised, even without being familiar with the term CPP.

Previous research suggests that long waiting times may lead to distrust in health services [19]. In our Western society, early detection, as being important for survival, is incorporated in how we understand cancer, to the degree that health services search for cancers with the highest incidence (i.e., breast, cervical and colorectal cancers) through screening in populations without symptoms [22]. Thus, it is the aim of the CPP to reduce waiting times could potentially lead to increased trust in health services if short waiting times are perceived as good care, but in the present study, patients had experienced changing pace through the CPP as a cause for worry. If populations are told that early action saves lives, being put in a fast lane CPP and then put on hold could be disturbing.

Therefore, it is important that patients receive sufficient information about CPP so they know what to expect with regards to waiting times through all phases of the CPP. Previous studies have shown that many patients prefer to know exactly when they will get their test results, while healthcare professionals rarely meet this preference [23]. However, patients’ preferences vary, and recommendations suggest that each patient should be asked about their information preferences [24]. When referring a patient to a CPP (i.e., before it is known whether the patient has cancer or not), healthcare professionals may want to save the patient from unnecessary anxiety and choose to avoid informing the patient in overly specific terms. For the patient, however, imprecise information could lead to the feeling of being unprepared and consequently to increased fear [25]. Our results are in line with this previous research suggesting that patients who are unaware of the time frames of a CPP, or CPP in general, could interpret a rapid response to indicate severity and urgency of their diagnosis.

### Methodological considerations

This is the first study of patient experiences of waiting times in CPP in Norway. The study’s strength was that patients from three CPPs and three hospital trusts from geographically diverse areas were included. The sample was a limitation to the study as only five men participated and waiting times and treatment options vary between the three forms of cancer. Furthermore, interviews were done retrospectively, and participants may have forgotten or reinterpreted their experiences from the CPP after receiving cancer treatment. Interviews by phone could be a barrier to relating personal patient experiences. Only individuals who received a cancer diagnosis were included in the study, and our results are not directly transferrable to understand the experiences of individuals who went through a CPP and were discharged without a diagnosis.

## Conclusions

Experiences with waiting times within CPP depend not only on the linear time frame for each step of the CPP but also on patients’ expectations. Patients are comforted by being within a structured CPP that ensures rapid diagnosis and start of treatment. CPPs still need to be improved to avoid delays, allow for adaptions to patient needs, and include adequate information to avoid stress among patients.

## Data Availability

The datasets generated and analysed during the current study are not publicly available due to ethical regulations but are available from the corresponding author on reasonable request.
